# The Effect of Qiweibaizhu Powder Crude Polysaccharide on Antibiotic-Associated Diarrhea Mice Is Associated With Restoring Intestinal Mucosal Bacteria

**DOI:** 10.3389/fnut.2022.952647

**Published:** 2022-07-08

**Authors:** Cuiru Li, Kang Zhou, Nenqun Xiao, Maijiao Peng, Zhoujin Tan

**Affiliations:** ^1^College of Chinese Medicine, Hunan University of Chinese Medicine, Changsha, China; ^2^College of Pharmacy, Hunan University of Chinese Medicine, Changsha, China

**Keywords:** QWBZP crude polysaccharide, antibiotic-associated diarrhea, intestinal mucosa, bacterial diversity, MiSeq

## Abstract

**Background:**

Qiweibaizhu powder (QWBZP) has been shown to be effective in treating antibiotic-associated diarrhea (AAD). Previous research has reported that plant polysaccharides can promote the growth of beneficial intestinal bacteria and inhibit the multiplication of pathogenic bacteria, thus effectively treating diarrhea. Here, we investigated the effect of QWBZP crude polysaccharide on the diversity of intestinal mucosal bacteria and their community structure composition in mice with AAD, and the aim of this study was to provide the scientific basis for the efficacy of QWBZP crude polysaccharide on diarrhea.

**Materials and Methods:**

Eighteen Kunming (KM) mice were randomly divided into the normal (mn) group, the model (mm) group, and the QWBZP crude polysaccharide treatment (ma) group, with six mice in each group. An AAD model was constructed using a mixed antibiotic solution and treated with gavage crude polysaccharide solution of QWBZP. The intestinal mucosa was extracted from the jejunum to the ileum of mice, and the metagenome was extracted and then analyzed using MiSeq sequencing to characterize the intestinal mucosal bacteria in mice.

**Results:**

The spleen and thymus indices of each group of mice had no significant differences. The Chao1 and ACE indices of the mn and mm groups were similar, the Simpson index was the largest and the Shannon index was the smallest in the mm group, and there was no significant difference in the diversity indices of all three groups. In the PCA and PCoA, the mn and ma group samples were both relatively concentrated with a high similarity of community structure. In contrast, the samples in the mm group were more scattered and farther away from the samples in the mn and ma groups, i.e., the community structure similarity within and between the groups was low. Compared with the mm group, the relative abundance of Proteobacteria, *Lactobacillus*, and the Firmicutes/Bacteroidetes (F/B) ratio in the ma group was decreased, while that of *Enterococcus* continued to increase. In the LEfSe analysis, there were significant differences in the characteristic bacteria in the mn, mm, and ma groups.

**Conclusion:**

The single crude polysaccharide component is not very effective in treating AAD, so the clinical efficacy of the QWBZP crude polysaccharide is subject to further investigation.

## Introduction

Healthy intestinal bacteria can produce organic acids through their metabolites, such as short-chain fatty acids, to maintain stool water content, repair and promote intestinal functions, lower intestinal pH, regulate the intestinal neuromuscular activity, and enhance intestinal peristalsis, thus promoting intestinal evolution and absorption functions, while effectively inhibiting the growth of spoilage bacteria in the intestine, improving the intestinal environment, and making stools soft and easy to excrete (1). However, this balance can be disrupted or compositionally disturbed under the stimulation of some external environment. When foreign intestinal pathogenic bacteria enter the organism, it will lead to acute diarrhea, transforming into chronic diarrhea if not treated in time (2). Long-term diarrhea will further aggravate the dysbiosis, forming a vicious cycle of persistent diarrhea and chronic diarrhea. Currently, antibiotics are widely used, which destroy pathogenic bacteria and kill a large number of normal bacteria, thus causing dysbiosis of the intestinal bacteria and increasing the number of conditionally pathogenic or drug-resistant bacteria, which are clinically known as antibiotic-associated diarrhea (AAD). Currently, antibiotics are the most common factor causing intestinal dysbiosis. The more antibiotics used, the broader the antibacterial spectrum, the longer the duration of use, and the younger the patient, the more likely they are to have intestinal dysbiosis (3). Almost all antibiotics can cause AAD, with a high incidence of diarrhea caused by lincomycin, cephalosporins, azithromycin, and ampicillin and a relatively low incidence of amino acid glycoside antibiotics (2). At the same time, the irrational use of antibiotics leads to significant changes in the structure of the intestinal bacteria and disrupts their metabolic capacity, which slows down the development of the microbial community, impairs the diversity and stability of the intestinal bacteria, and increases the risk of diseases such as inflammatory bowel disease, obesity, diabetes, wheezing, and allergies (4, 5).

Dysbiosis diarrhea can be regarded as spleen deficiency diarrhea, so the principle of treatment for AAD in Chinese medicine is to invigorate the spleen, and QWBZP is a more frequently used herbal formula. It was initially known as Baizhu powder, consisting of Sijunzi decoction (ginseng, Poria cocos, Atractylodes macrocephala, and licorice) with Costus roots, Agastache, and Pueraria. It was created by Qianyi, the originator of pediatrics in the Northern Song Dynasty, and is recorded in “Key to Therapeutics of Children's Diseases,” which has the effect of invigorating the spleen and producing fluid, promoting Qi, and eliminating swelling. Experimental studies have shown that the treatment of colon cancer (6) and ulcerative colitis (7) with Sijunzi decoction both resulted in a rise in the number of *Bifidobacterium* and *Lactobacillus* and a decrease in Enterobacteriaceae and *Enterococcus* in the intestine of mice. Therefore, it is believed that Sijunzi decoction can support the growth of normal bacteria and regulate the distribution of intestinal bacteria. Moreover, Agastache extract (8) and volatile oil components (8), Costus roots extract (9) and volatile oil components (10), as well as crude polysaccharides of Pueraria (11) and puerarin (12), have direct bacteriostasis and bactericidal effects *in vitro*. Thus, it can be assumed that QWBZP, a derivative of Sijunzi decoction, also has sound clinical effects on AAD. In an experiment in which mice were molded with a mixture of antibiotics and intervened with ultra-micro-powder Qiweibaizhusan (46), the count of total intestinal bacteria, *E. coli*, intestinal lactic acid bacteria, and intestinal yeast was significantly increased, which was regulated to reach a new equilibrium level while causing a significant improvement in diarrheal symptoms. A large number of experimental studies (14–16) have shown that QWBZP can improve diarrhea by restoring the diversity of intestinal bacteria and regulating the structure of intestinal bacteria in mice, thereby repairing the mucosal barrier and protecting the intestine, revealing the mechanism of action of QWBZP in the treatment of AAD. Our research group has conducted a dynamic study on the intestinal flora of the established AAD model with QWBZP. Using microbiological and enzymatic techniques, we investigated the effects of traditional decoction of QWBZP (17–19), ultra-micro-powder Qiweibaizhusan (13), and the combination of QWBZP with sucrose (20, 21) or yeast (22, 23) on intestinal microorganisms and enzymes, to preliminarily reveal the vital herbal components of the efficacy of QWBZP and the mechanism of the effectiveness of ultramicroscopic herbal medicines.

In recent years, their biological activities have been explored with continuous research on plant polysaccharides. Polysaccharides extracted from different herbs have received wide attention for their important biological activities, such as antioxidant (scavenging free radicals and regulating related enzyme activities), antitumor (enhancing body immune function and affecting tumor cell metabolism), hypolipidemic (inhibiting lipase), hypoglycemic (regulating islet cells and regulating insulin), immunomodulatory, antiviral, antiradiation, anticoagulant, antiulcer, and bacteriostasis, with low toxic side effects (24, 25). Herbal polysaccharides can promote the proliferation of beneficial intestinal bacteria; while fermenting plant polysaccharides in the intestine to produce organic acids to lower the intestinal pH, harmful bacteria in the intestine cannot effectively use plant polysaccharides; therefore, they have an inhibitory effect on the growth and reproduction of pathogenic bacteria (13). In addition, Shen et al. (26) showed through their study that herbal polysaccharides can treat diarrhea by modulating the body's immune response. It has been demonstrated that herbal polysaccharides have essential medicinal value, providing evidence for their critical role in medical applications. Therefore, the study on the effect of QWBZP crude polysaccharide on intestinal bacteria with diarrhea can provide a particular reference significance for future research, development, and application of herbal polysaccharides as functional foods and modern medical treatments.

## Materials and Methods

### Animals

Eighteen 1-month-old specific pathogen-free (SPF) Kunming (KM) mice (nine males and nine females) weighing 18 g−22 g were purchased from Hunan Slaccas Jingda Laboratory Animal Company (Hunan, China) with license number SCXK (Xiang) 2016–2002. All experiments and procedures involving animals were performed according to the protocols approved by the Institutional Animal Care and Use Committee of the Hunan University of Chinese Medicine.

### Feed

The mice feed was provided by the Experimental Animal Center of Hunan University of Traditional Chinese Medicine. The main indicators of nutrients include moisture, crude protein, crude fiber, crude fat, crude ash, calcium, total phosphorus, lysine, methionine, and cystine.

### Medicine

According to the Chinese Pharmacopeia 2020, QWBZP was composed of 6 g of ginseng (Shanxi), 10 g of Poria cocos (Yunan), 6 g of Costus roots (Yunnan), 10 g of Agastache (Guangdong), 10 g of Pueraria (Hunan), 10 g of Atractylodes macrocephala (Zhejiang), and 3 g of licorice (Neimong), and the same batch of herbs was purchased from the First Affiliated Hospital of Hunan University of Traditional Chinese Medicine. The above is the amount of one dose of soup.

### Reagents and Preparation

A mixed antibiotic solution of gentamicin sulfate injection (Yichang Renfu Pharmaceutical Co., Ltd., State Drug Quantifier H42022058, product batch number: 5120106) and cephradine capsule (Suzhou Sinochem Pharmaceutical Industry Co., Ltd., product batch number: 110804) was prepared to make a concentration of 62.5 g/L [i.e., 6 gentamicin (2 mL/branch) + 3 cephalosporins (0.25 g/grain)] and stored at 4°C for the following experiment (27). Anhydrous glucose, anhydrous ethanol, sulfuric acid, and phenol were analytical reagents purchased from Sinopharm Chemical Reagent Co. (28).

The medicine was weighed according to the above ratio, and cold water was added to soak the surface of the medicine for 30 min. The medicine was decocted with a large flame until it boiled and then with a soft flame. The decoction time was 20–30 min. The medicine was successively decocted two times. The liquid medicine decocted two times was mixed to make Qiweibaizhu decoction. Filtration was repeated three times with gauze. The filtrate was combined, concentrated by evaporation, and cooled. Three times the volume of anhydrous ethanol was added to a final concentration of 75% ethanol. The solution was stirred well and precipitated overnight at 4°C. The precipitate was washed three times with 75% ethanol, dried to constant weight, ground into powder, and sealed in a desiccator. Before use, the crude polysaccharide solution was dissolved in sterile water. The total sugar in the QWBZP crude polysaccharide was measured by the phenol–sulfuric acid method at 0.028 g/mL (28).

### Modeling and Treatment

**Figure 1:**
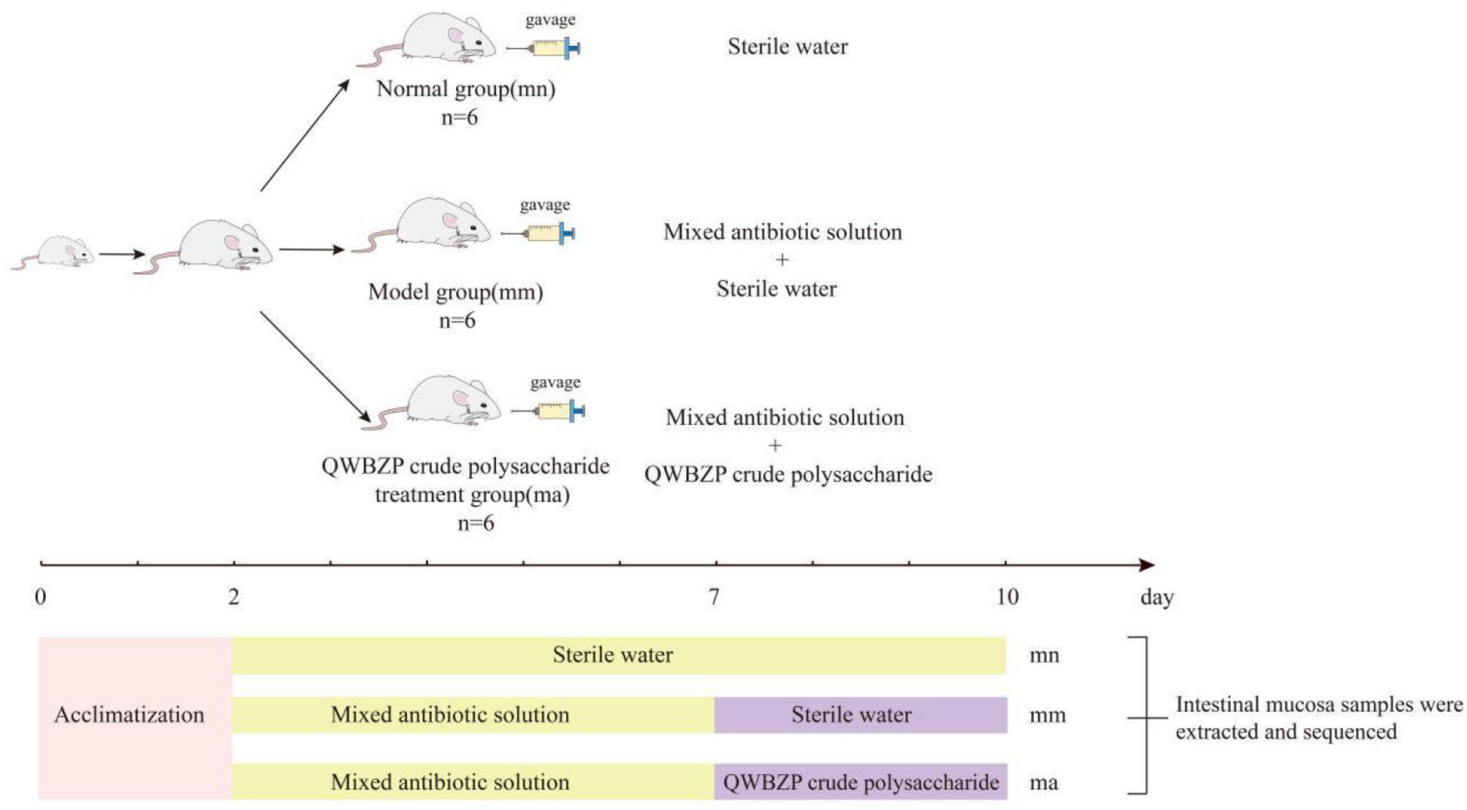
Experimental flowchart.

After 2 days of adaptive feeding (room temperature 23–25°C, relative humidity 50–70%, clean and quiet), 18 KM mice (equal numbers of males and females) were randomly divided into normal (mn) group, model (mm) group, and QWBZP crude polysaccharide treatment (ma) group, with six mice in each group. To induce diarrhea, mice in both the mm and ma groups were administered an antibiotic mixture of gentamicin sulfate and cephradine (23.33 mL·kg^−1^·d^−1^). Correspondingly, mice in the mn group were gavaged with sterile water, 0.35 mL two times per day for 5 days. When diarrhea symptoms were induced (declined activity, arched back trembling, specifically watery stool, curled up, and poor appetite) (29), mice in the ma group were gavaged with QWBZP crude polysaccharide, 0.35 mL two times per day for 3 days. Correspondingly, mice in both the mn and mm groups were gavaged with sterile water. The procedure is shown in Figure 1.

### General Features

The general status of each group of mice before and after modeling and after drug administration was observed separately, including body weight, food intake, activity, mental status, stool, and the presence of prolapse.

### Measurement of Organ Index

Each mouse was weighed. After the mice were sacrificed using cervical vertebra dislocation on a sterile operation platform, the intact spleen and thymus were removed, and the attached surface fascia and the adipose tissue were also removed. Then, blood was blotted from the visceral surface with a filter paper and weighed separately, and the spleen and thymus indices were calculated: visceral index = visceral weight (mg)/body weight (g).

### Extraction of Mice Intestinal Mucosa

After mice were sacrificed using cervical vertebra dislocation on a sterile operation platform, the jejunum to ileum segment was taken as the test specimens. The intestine was dissected after squeezing out the contents of the chymus, and then, the intestinal wall was washed with saline. The intestinal mucosa was scraped with coverslips, and two times the weight of saline was added. It was homogenized at low speed for 1 min and then centrifuged at 3,000 r/min for 10 min. The supernatant was taken for subsequent gene extraction. Finally, intestinal mucosa samples from two mice (one male and one female) in every group were selected and immediately frozen at 4 °C for the following experiment (15, 30).

### Metagenome Extraction

Metagenome DNA of intestinal mucosal microorganisms was extracted according to our previous methods (31) with the following processes: acetone washing, lysozyme wall breaking, proteinase K denaturation, SDS lysis, CTAB treatment, and phenol/chloroform extraction to obtain high-quality DNA.

### PCR Amplification and MiSeq Metagenome Sequencing

The V3+V4 variable region of bacterial 16S rDNA was amplified using the extracted DNA as a template with primers 338F (5'-ACTCCTACGGGAGGCAGCA-3') and 806R (5'-GGACTACHVGGGTWTCTAAT-3'). The PCR products of the same samples were mixed and detected by 2% agarose gel electrophoresis, purified using the AxyPred Gel Extraction Kit (Axygen Scientific Inc., Union City, CA, USA), and quantified by QuantiFluor™ -ST Blue Fluorescence Quantification System (Promega), after which the corresponding proportion was mixed according to the sequencing volume requirement of each sample. A TransStart Fastpfu DNA Polymerase 20-μL reaction system was used including: 2.0 μL of 10 × buffer, 2.0 μL of 2.5 m MdNTPs, 0.8 μL of forward primer (5 μmol/L), 0.8 μL of reverse primer (5 μmol/L), 0.2 μL of Taq polymerase, 0.2 μL of BSA, 10 μL of template DNA, and 20 μL of ddH2O. The amplification conditions were as follows: pre-denaturation at 95°C for 3 min, followed by 29 cycles at 95°C for 30 s, annealing at 55°C for 30 s, extension at 72°C for 45 s, and then 72°C for 10 min (32).

The PCR products were then sequenced by the Illumina MiSeq sequencing platform (Illumina, San Diego, CA, USA). MiSeq metagenomic sequencing was completed by Wuhan Fraser Genetic Information Co., Ltd.

### Bioinformatics and Statistical Analysis

#### OTU Division and Classification Status Identification

OTU clustering of non-repetitive sequences (excluding single sequences) was performed at 97% similarity using Usearch 7.1 software. The taxonomic analysis of OTU representative sequences at 97% similarity was performed on the QIIME platform using an RDP classifier Bayesian algorithm and compared to the SILVA (33) database to obtain the species classification information corresponding to each OTU.

#### Alpha Diversity Analysis

The Chao1, ACE, Simpson, and Shannon indices were calculated and analyzed using Mothur (version 1.30.1) (34).

#### Beta Diversity Analysis

It is mainly used to compare the differences between different samples, and the commonly used distance algorithms are Jaccard, Bray–Curtis, UniFrac, etc. After obtaining the distance matrix, the ranking analysis was performed and visualized by PCA (35), PCoA (36), etc., to analyze the differences in community composition between samples or subgroups.

#### LEfSe Analysis

Linear discriminant analysis (LDA) was performed on samples according to different grouping conditions using LEfSe analysis software (37) based on taxonomic composition to identify communities or species with a significant differential impact on sample delineation.

#### Statistical Analysis

The measurement data obtained from each group were expressed as mean ± standard deviation (x±s), one-way ANOVA was performed using SPSS 25.0 software, and the data were compared between two groups using independent samples t-test, with *p* < 0.05 indicating a significant difference.

## Results

### General Features

Before modeling, the mice had normal food intake and response, smooth hair, and dry black feces pellets, which did not stick to the hands when squashed. From day 2 to day 5 after modeling, the mice in the mm group had diarrhea one after another. On day 5, all the mice in the mm group had noticeable diarrhea, with clinical manifestations of thin and soft stools, watery yellow stools, and individual mice had prolapse. Their food intake decreased and showed a pile-up phenomenon. Their hair lost its luster, while the mice in the mn group did not show any obvious abnormality, indicating that the AAD model was successfully prepared. After treatment with QWBZP crude polysaccharide, compared with the mice in the mn group, the mice in the mm group had better dilute and soft stools than before treatment, the stools were granular and stuck to the hands after squashing, the amount of food intake was reduced, and they liked to curl up their bodies, and the hair was dry. The mice in the ma group had granular feces, flexible activities, dense and lustrous hair, and normal feeding and urine intake, suggesting that QWBZP crude polysaccharide was effective in treating AAD in mice and improving their feeding condition and other related symptoms.

### Effect of QWBZP Crude Polysaccharide on Visceral Indices of AAD Mice

**Figure 2:**
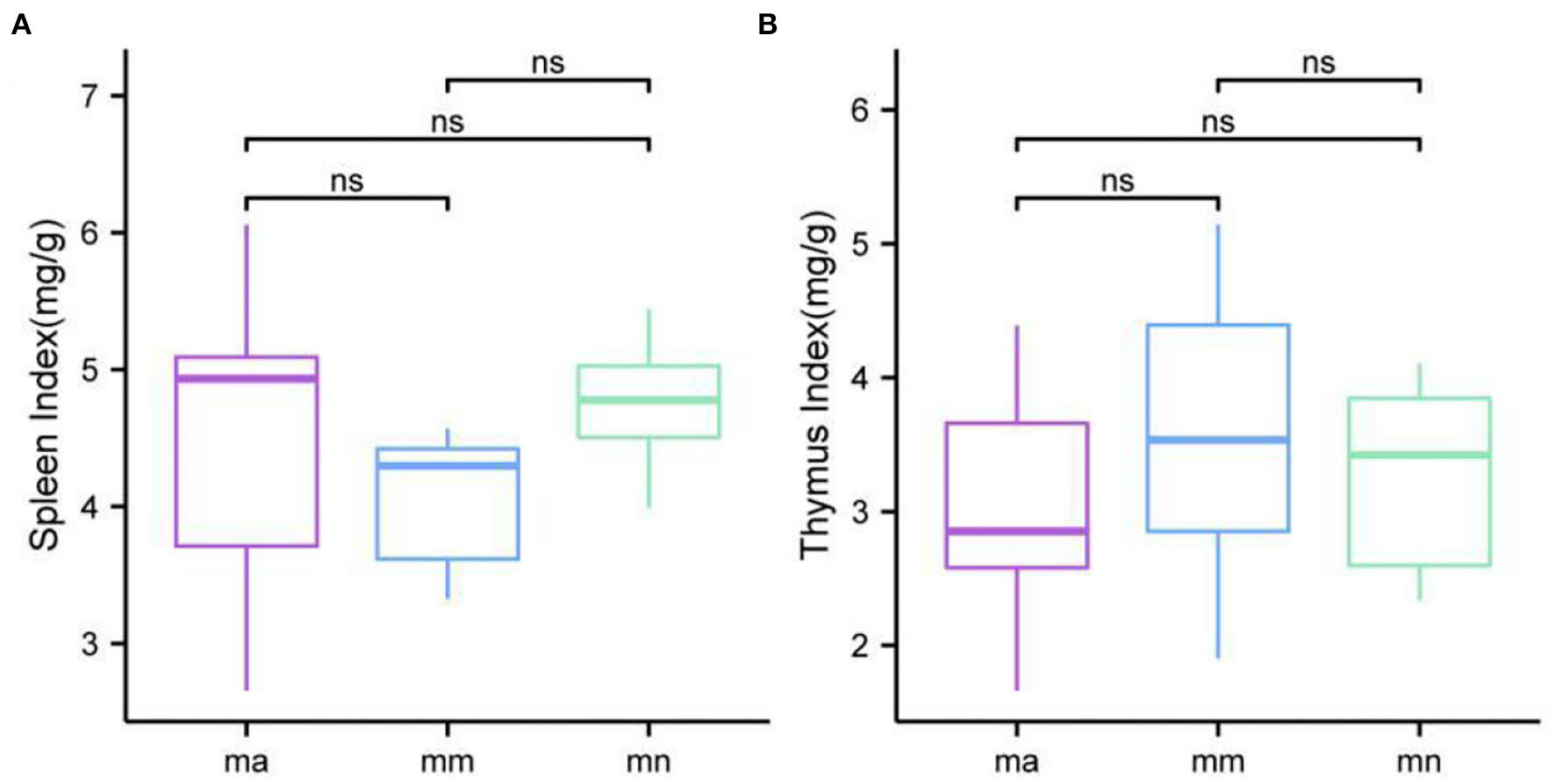
Comparison of the spleen index and the thymus index. Box plots of **(A)** spleen index and **(B)** thymus index.

As shown in Figure 2, the spleen index of mice in the mm group was reduced compared with the mn group, but there was no significant difference. Compared with the mm group, the spleen index of mice in the ma group was increased, but there was no significant difference. There was no significant difference in the thymus index of mice in all groups.

### Effect of QWBZP Crude Polysaccharide on Bacterial OTU Number and Dilution Curve in the Intestinal Mucosa of AAD Mice

**Figure 3:**
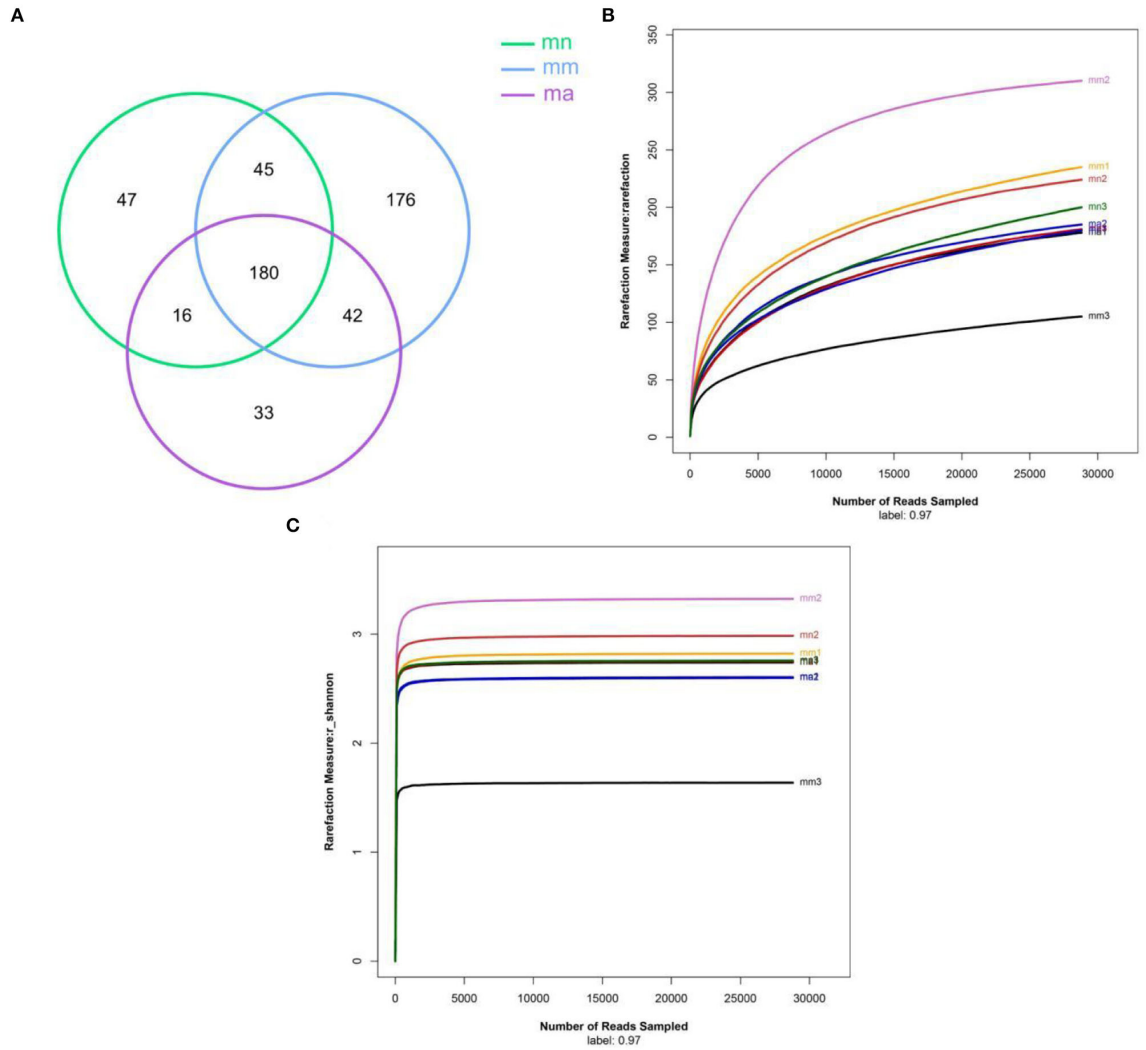
**(A)** Venn diagram: the distribution of the number of intestinal mucosal bacterial OTUs in each group of samples. Green, blue, and purple represent the mn, mm, and ma groups, respectively. **(B)** Dilution curve of intestinal mucosal bacteria in each group of samples. Horizontal coordinate: the amount of randomly selected sequencing data; vertical coordinate: the number of observed OTUs. **(C)** The Shannon–Wiener curve of intestinal mucosal bacteria for each group of samples. mn: normal group; mm: model group; ma: QWBZP crude polysaccharide treatment group.

OTU Venn diagram analyzed the similarity and overlap of community structures of different samples and visualized the similarity and uniqueness of samples at the OTU level. The analysis results are shown in Figure 3A. The total number of OTUs for the three experimental groups was 180, and there were 288, 443, and 271 OTUs found in the mn, mm, and ma groups, respectively. It showed that antibiotic modeling increased the OTU number of intestinal mucosal bacteria in mice. After treatment with QWBZP crude polysaccharide, the OTU number of intestinal mucosal bacteria in mice decreased significantly and approached the mn group. According to the dilution curves of the mn, mm, and ma groups (Figure 3B), it can be seen that the species richness of the mn and ma groups was similar. Compared with the mm group, the mn and ma groups differed significantly, and there were individual differences. The dilution curves smoothed out for all three experimental groups, indicating that the sequencing data could cover the vast majority of species in the sample and that more data volume would only generate a small number of new OTUs.

### Effect of QWBZP Crude Polysaccharide on Alpha Diversity in the Intestinal Mucosa of AAD Mice

**Figure 4:**
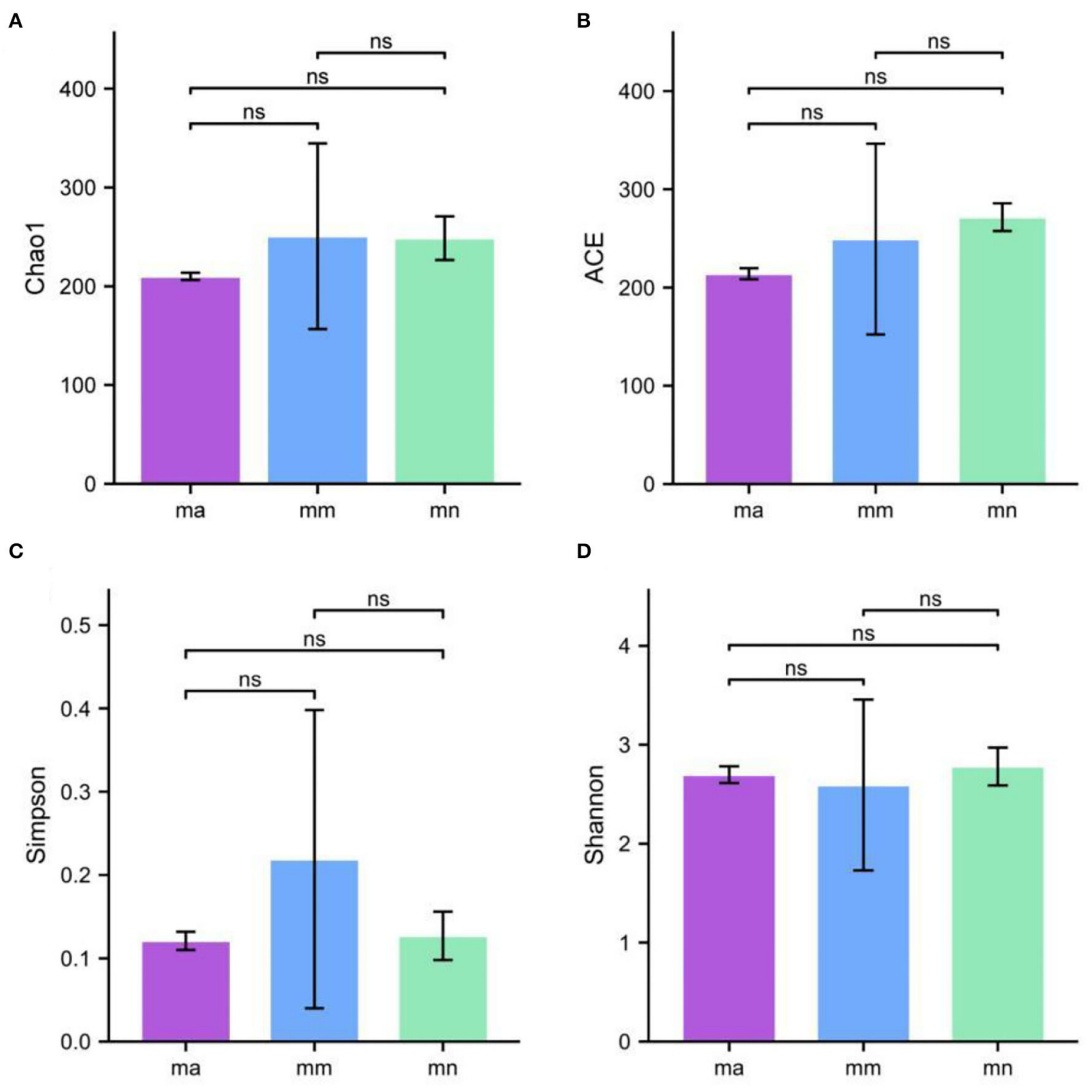
Effect of QWBZP crude polysaccharide on the alpha diversity index of mice intestinal mucosal bacteria. **(A)** Chao1. **(B)** ACE. **(C)** Simpson. **(D)** Shannon.

Alpha diversity describes the biodiversity within a particular region or ecosystem, i.e., assessing the biodiversity of a given sample, and is generally characterized by calculating a diversity index based on species richness or evenness. The Chao1 and ACE indices are often used to estimate the total number of community species: the larger the index, the more the total number of community species. The Shannon and Simpson indices consider both species richness and evenness; they can objectively reflect the diversity of community species. The larger the value of the Simpson index, or the smaller the value of the Shannon index, the lower the community diversity. From Figure 4, it is clear that the Chao1 index was in the descending order of the mm group, the mn group, and the ma group; the rank of the ACE index from high to low was ranked as mn, mm, and ma; for the Simpson index, the ranking was as follows: mm, mn, and ma; and for the Shannon index from high to low was ranked as mn, ma, and mm, all of which were not statistical difference (*p* > 0.05). The results suggest that the QWBZP crude polysaccharide can affect the diversity of intestinal mucosal bacteria in mice. The Shannon–Wiener curve (35) was constructed using the microbial diversity index for each sample at different sequencing depths as a reflection of the microbial diversity of each sample at different sequencing quantities. As shown in Figure 3C, the Shannon–Wiener curves of the three experimental groups tended to be flat, indicating that the amount of sequencing data was large enough to reflect the majority of microbial information in the samples.

### Effect of QWBZP Crude Polysaccharide on Beta Diversity in the Intestinal Mucosa of AAD Mice

**Figure 5:**
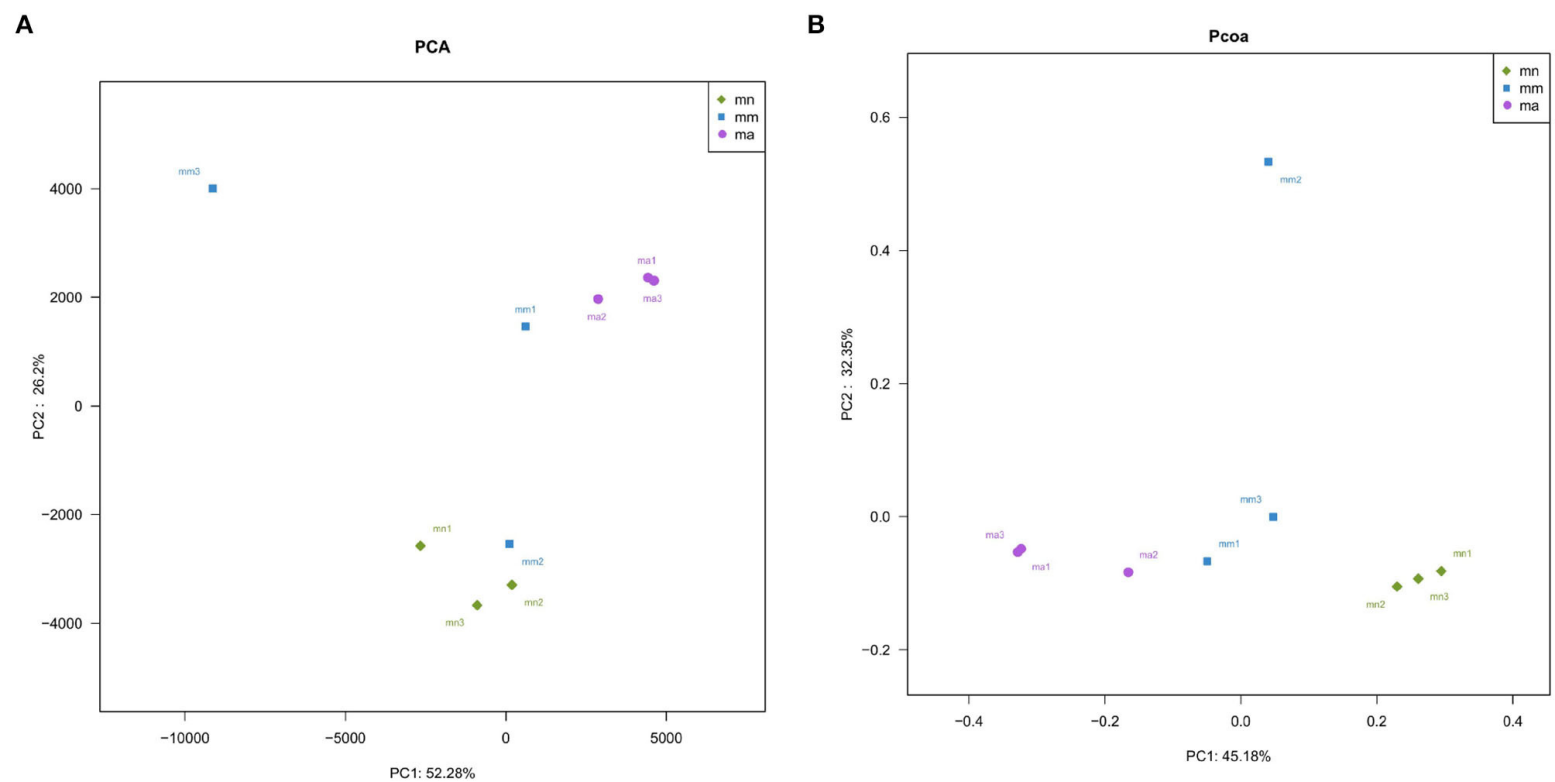
Effect of QWBZP crude polysaccharide on the beta diversity of mice intestinal mucosal bacteria. Green, blue, and purple represent the mn, mm, and ma groups, respectively. Two-dimensional sorting by **(A)** PCA and **(B)** PCoA.

Beta diversity describes the differences in species composition between habitat communities, that is, comparing differences between samples. Principal component analysis (PCA), a simplified data analysis technique, can reflect the differences and distances between samples by analyzing the composition of different samples OTU (97% similarity). PCA uses variance decomposition to reflect the differences in multiple datasets of data on a two-dimensional coordinate plot. The axes take the two eigenvalues that best reflect the variance values. The more similar the sample composition, the closer the distance reflected in the PCA plot (35). As shown in Figure 5A, the principal component variable 1 was 52.28% and the principal component variable 2 was 26.2%. Samples in the mn group were relatively concentrated, while samples in the mm group were more dispersed. The distance between the mn and mm groups was farther, indicating that antibiotic modeling had a significant effect on the structure of the intestinal mucosal bacteria. After treatment with QWBZP crude polysaccharide, samples ma1, ma2, and ma3 were more concentrated. Principal coordinate analysis (PCoA), a visualization method to study data similarity or variability, can be used to study the similarity or variability in the composition of sample communities (36). As shown in Figure 5B, samples in the mn and ma groups were more concentrated, while samples in the mm group were relatively dispersed. The PCA and PCoA indicate that QWBZP crude polysaccharide can effectively restore the structure of intestinal mucosal bacteria in mice in the mm group.

### Effect of QWBZP Crude Polysaccharide on the Relative Abundance of Intestinal Mucosal of AAD Mice

**Figure 6:**
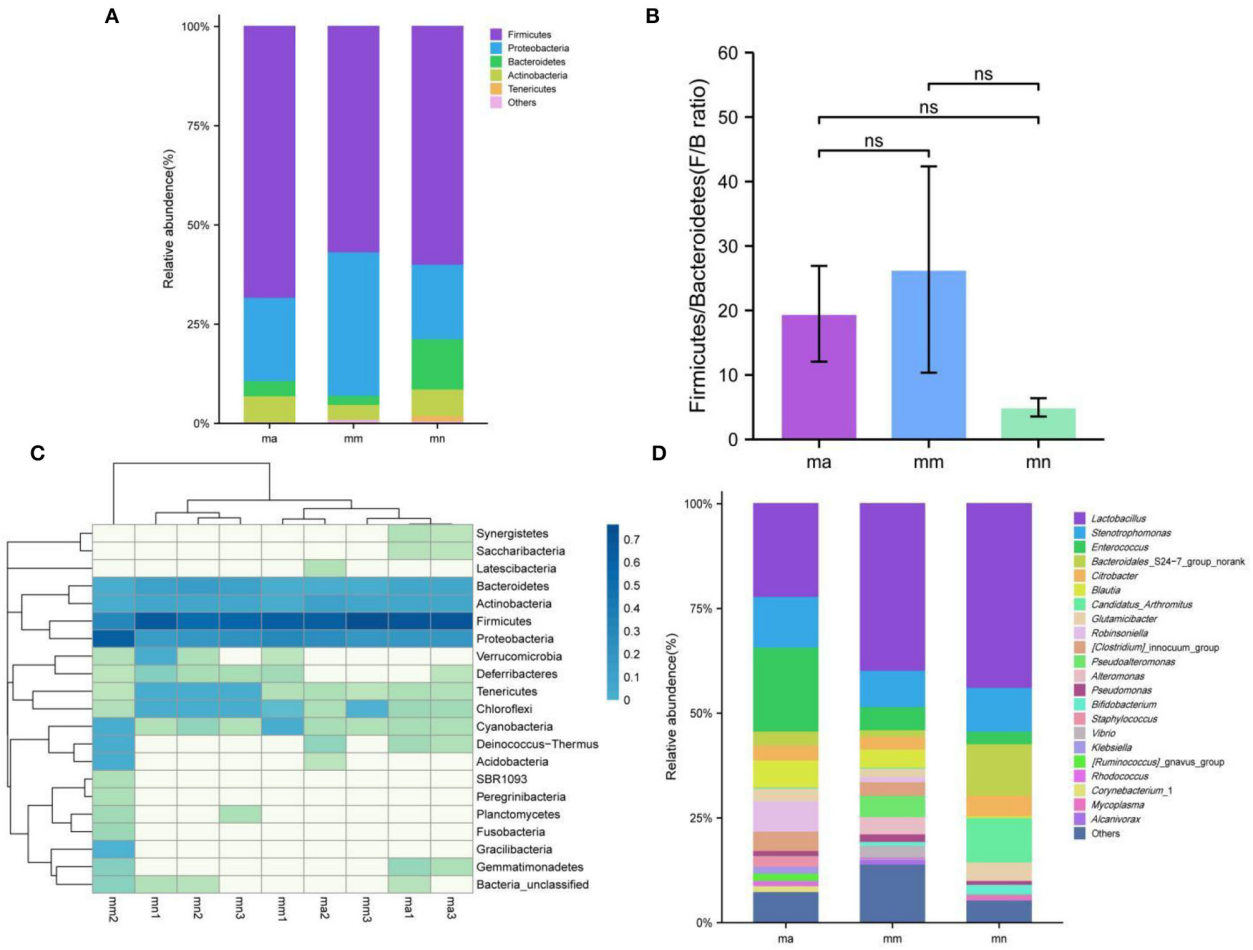
Effect of QWBZP crude polysaccharide on the relative abundance of intestinal mucosal bacteria. mn, normal group; mm, model group; ma, QWBZP crude polysaccharide treatment group. **(A)** Relative abundance at the phylum level. **(B)** Histogram of Firmicutes/Bacteroidetes ratio. **(C)** Heat map of relative abundance at the phylum level. **(D)** Relative abundance at the genus level.

Figure 6A shows the relative abundance of intestinal mucosal bacteria in mice at the phylum level, in which five taxa, namely, Firmicutes, Proteobacteria, Bacteroidetes, Actinobacteria, and Tenericutes, were the dominant phylum and accounted for a large proportion. Compared with the mn group, the relative abundance of Firmicutes (*P* > 0.05), Bacteroidetes (*P* < 0.05), Actinobacteria (*P* > 0.05), and Tenericutes (*P* < 0.05) decreased, while that of Proteobacteria (*P* > 0.05) increased in the mm group. Compared with the mm group, the relative abundance of Firmicutes (*P* > 0.05), Bacteroidetes (*P* > 0.05), Actinobacteria (*P* < 0.05), and Tenericutes (*P* > 0.05) increased, while that of Proteobacteria (*P* > 0.05) decreased after treatment with QWBZP crude polysaccharide. Among them, the relative abundance of Firmicutes (*P* > 0.05) was higher than that of the mn group, that of Proteobacteria (*P* > 0.05) was not significantly different from that of the mn group, and that of Bacteroidetes (*P* < 0.05), Actinobacteria (*P* > 0.05), and Tenericutes (*P* < 0.05) was lower than that of the mn group. Figure 6B shows the ratio of Firmicutes and Bacteroidetes (F/B ratio). Compared with the mn group, the F/B ratio increased in the mm group but decreased after treatment with QWBZP crude polysaccharide.

A heat map can simultaneously present information on community species composition and abundance and visually reflect the similarities and differences in community composition of different samples or subgroups through color changes, and cluster analysis was also performed based on the similarity in abundance between species or samples. The horizontal axis of the heat map indicates the abundance of clustering similarity between samples, and it can be seen from Figure 6C that among the detected phyla, both the mn and ma groups can be better clustered into one category, and the three samples in the mm group are more scattered, probably because there are individual differences among the three samples of mm1, mm2, and mm3 mice, resulting in differences in the changes of intestinal mucosal bacteria after the administration of antibiotics to mice. The vertical axis of the heat map indicates the abundance of clustering similarity among species, which were clearly separated by a relative abundance, and the clades with high abundance were high in all groups, but those with low abundance were low in all groups.

Figure 6D shows the relative abundance of intestinal mucosal bacteria in mice at the genus level. *Enterococcus, Lactobacillus*, and *Stenotrophomonas* are the dominant genera with a large proportion. Compared with the mn group, the relative abundance of *Stenotrophomonas* (*P* > 0.05), *Lactobacillus* (*P* > 0.05), *Candidatus_Arthromitus* (*P* < 0.05), and *Bacteroidales*_S24-7_group_norank (*P* < 0.05) decreased and that of *Enterococcus* (*P* > 0.05) increased in the mm group. Compared with the mm group, after treatment with the QWBZP crude polysaccharide, the relative abundance of *Enterococcus* (*P* < 0.05) continued to increase, while that of *Stenotrophomonas* (*P* > 0.05) increased and reached the level of the mn group; that of *Bacteroidales*_S24-7_group_norank (*P* > 0.05) increased, but was still lower than that of the mn group; that of *Candidatus_Arthromitus* (*P* > 0.05) was not significantly different from that of the mn group; and that of *Lactobacillus* (*P* > 0.05) was decreased.

### Effect of QWBZP Crude Polysaccharide on the Characteristic Intestinal Mucosa Bacteria of AAD Mice

**Figure 7:**
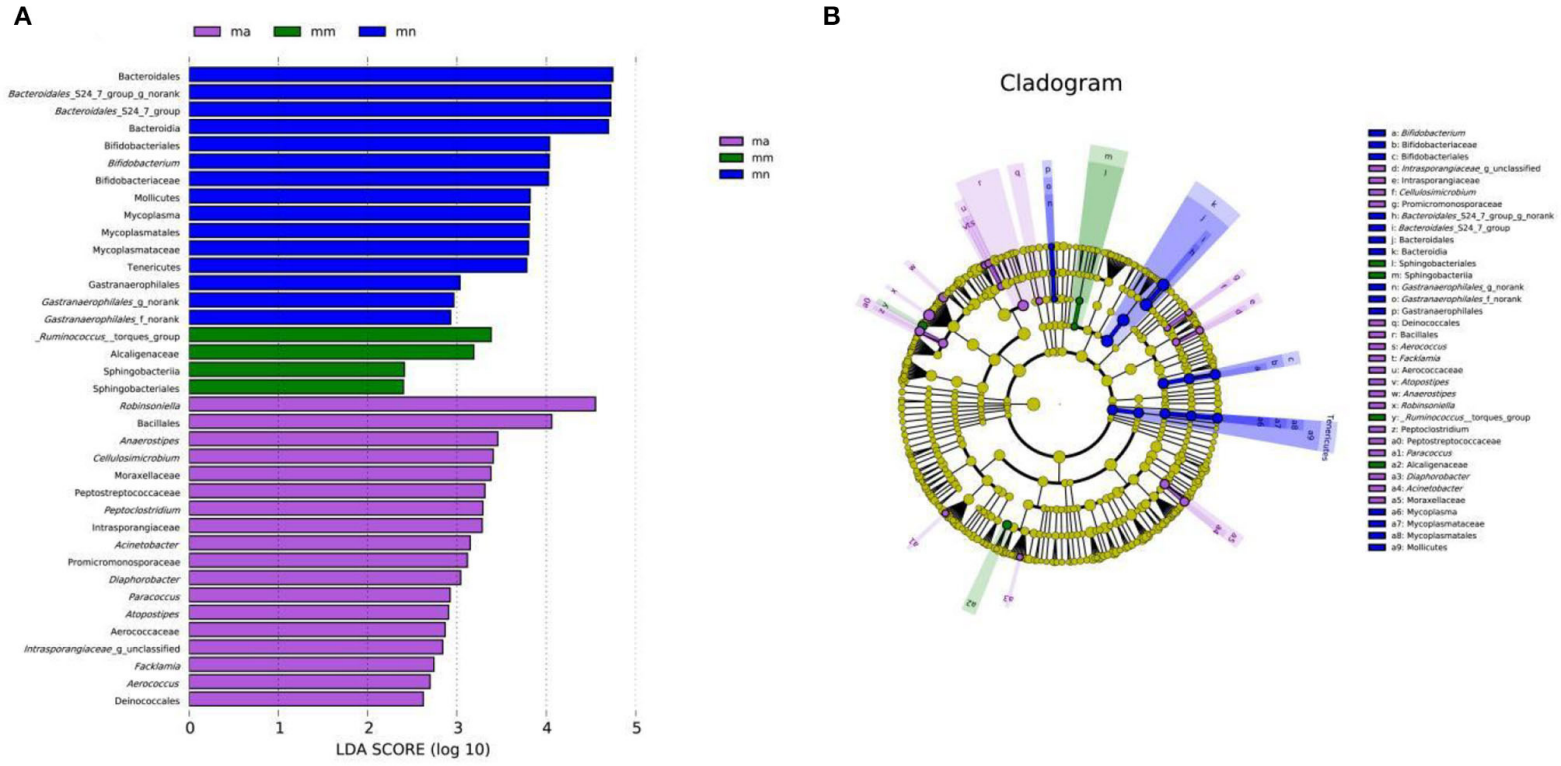
Effect of QWBZP crude polysaccharide on the characteristic intestinal mucosal bacteria. Green, blue, and purple represent the mn, mm, and ma groups, respectively. **(A)** LDA score plot. **(B)** LEfSe analysis.

As shown in Figure 7A, there were 15, 4, and 18 dominant groups in the mn, mm, and ma groups, respectively. The main characteristic bacteria in the mn group were Bacteroidales, *Bacteroidales*_S24-7_group_g_norank, and *Bacteroidales*_S24-7_group, Bacteroidaceae; _*Ruminococcus*_torgues_ group, Alcaligenaceae, were the most prominent bacteria in the mm group; and *Robinsonella*, Bacillales, were significantly enriched in the ma group. The results showed significant differences in the intestinal mucosal bacteria and characteristic bacteria between the three groups. The taxonomy shown in Figure 7B represents the structure of the gut microbes and their main bacteria, offering the most significant taxonomic differences between the three groups. This is further evidence of the differences in the structure of the gut microbes in the mn, mm, and ma groups.

## Discussion

The intestine is the organ with the most significant contact area between the external environment and the host's internal environment. The intestinal mucosal barrier assists the body in maintaining the integrity of the intestine and immune homeostasis through dynamic changes. Functionally, the intestinal mucosal barrier can be classified into mechanical, chemical, biological, and immune barriers. The intestinal flora plays an essential role in regulating the four major barriers of the intestinal mucosa. A functionally intact biological barrier of the intestinal mucosa plays an important role in maintaining the health of the organism, while changes in the type and number of intestinal mucosal microorganisms accompanied by pathological changes such as destruction of intestinal mucosal tissues, decreased expression of tight junction proteins, and increased permeability of the intestinal mucosa can affect the barrier function of the intestinal mucosa (38). In the case of long-term antibiotic abuse, it can lead to the reduction in or even complete elimination of a certain percentage of the intestinal flora, the overproduction of certain new strains of bacteria, or the promotion of the mutation of antibiotic-resistant genes in the intestinal flora, raising the risk of infestation by potentially pathogenic bacteria and causing flora dysbiosis in severe cases. In turn, the alteration in flora structure can further weaken the mucin, cytokines, and antimicrobial peptides produced by intestinal epithelial cells, which in turn weakens the intestinal epithelial barrier and leaves the intestine vulnerable to infection (2). In clinical treatment, it is found that multiple herbs in combination have certain effects on restoring intestinal function and protecting the intestinal mucosal barrier. Multiple components in compound herbs can participate in establishing intestinal mucosal immune function and protecting the intestinal barrier by increasing immune cell activation, regulating the ratio of lymphocytes, promoting sIgA production in intestinal tissues, regulating immune molecules and immune cells of the body to regulate intestinal cytokine expression, and affecting intestinal flora homeostasis (39). Related studies have shown that QWBZP can effectively increase intestinal sIgA content, regulate the expression of anti-inflammatory factors, reduce intestinal inflammation, repair damaged intestinal mucosal immune tissues, and minimize dysbiosis diarrhea (40, 45). Chinese herbal polysaccharides can play a role in repairing intestinal mucosal damage and promoting intestinal mucosal immunity by affecting the mechanical barrier of the intestinal mucosa and the secretion of immune cells, sIgA and cytokines, and intestinal symbiotic flora (41).

Combined with the results of this experiment, we found no significant differences in the spleen and thymus indices of each group of mice. The visceral index is a preliminary indicator of the immune function of the organism. The changes in the spleen and thymus indices can visually reflect the changes in the organism and cellular immunity level. They can roughly estimate the strength of immune function, but it is a crude and lagging index. The results of this experiment showed that the QWBZP crude polysaccharide could improve the spleen index, but could not improve the thymus index. This does not mean that it has no noticeable effect on the immune function of mice; after all, this is a rough index, which should be analyzed and discussed in conjunction with other results. Microbial diversity is studied in community ecology, and the alpha diversity analysis of samples can reflect the abundance and diversity of microbial communities. In this experiment, the Simpson index was the largest and the Shannon index was the smallest in the mm group, suggesting that antibiotic modeling had a certain inhibitory effect on the community diversity of intestinal mucosal bacteria. After treatment with QWBZP crude polysaccharide, the Simpson index and Shannon index were close to the level of the mn group, suggesting that QWBZP crude polysaccharide had a certain restoring effect on the community diversity of intestinal mucosa. The Chao1 and ACE indices of the mn and mm groups were similar, and there was no significant difference in the diversity indices of all three groups. It may be that mucosal bacteria have a strong self-regulatory effect and can recover naturally after a short-term antibiotic intervention. Our previous study (42) showed that antibiotic molding disrupts the microecological balance in the intestine and decreases the diversity of the intestinal bacteria, which may be related to the “extinction” effect of gavage antibiotics on some bacterial species in the intestine, or the inability of some intestinal resident bacteria to colonize and survive in the intestine by altering the mucosa of the intestinal wall or the intestinal environment. In terms of OTU count, the OTU number in the mm group (443) was significantly higher than that in both the mn (288) and ma (271) groups. Combined with the dilution curve, it can be seen that the richness of species in the ma group was close to that in the mn group, indicating that QWBZP crude polysaccharide helped restore intestinal diversity mucosal bacteria in mice to the normal level. It is presumed that QWBZP crude polysaccharide positively affects the richness, diversity, and microbial community of intestinal mucosal bacteria in antibiotic-molded mice. In the PCA, the samples in the mn and ma groups were both relatively concentrated and had high community structural similarities. In contrast, the samples in the mm group were more scattered and farther away from the samples in the mn and ma groups, that is, the community structural similarity within and between groups was low, there were noticeable structural differences, and the PCoA also yielded similar conclusions. It can be speculated that the antibiotic disrupted the intestinal mucosal bacterial structure, suggesting that the treatment with QWBZP crude polysaccharide can restore the intestinal mucosal bacterial structure to the normal level.

By comparing the changes in the abundance of intestinal mucosal bacteria in three experimental groups, we can further understand how QWBZP crude polysaccharide altered the intestinal microbial environment. In general, Firmicutes and Bacteroidetes dominate the intestinal microbial community at the phylum level, while the abundance of Proteobacteria and Actinobacteria is lower. The increasing number of Proteobacteria or F/B ratio would lead to an imbalance or instability in the structure of the intestinal microbial community (43). This study showed that compared with the mn group, the number of Proteobacteria in the mm group increased significantly and the F/B ratio also increased significantly, suggesting that antibiotic modeling led to an imbalance in the intestinal microecological environment. After treatment with QWBZP crude polysaccharide, the number of Proteobacteria was restored to a level close to that of the mn group. The F/B ratio also decreased but was still higher than the mn group. It can be speculated that QWBZP crude polysaccharide is beneficial to the recovery of the main bacteria of the intestinal mucosa, and the F/B ratio was still higher than that of the mn group, which was probably because the mice were still in the recovery stage. Compared with the mn group, the relative abundance of *Lactobacillus* showed a decreasing trend at the genus level, and *Enterococcus* continued to increase in the ma group. *Lactobacillus* is essential beneficial bacteria in the human intestine, which can improve intestinal microecology by inhibiting the growth of harmful microorganisms (44). Still, in the treatment of AAD with QWBZP, multiple components of the compound herbal medicine play an essential role in intestinal microecology at the same time (13), while in this experiment, only the crude polysaccharide in QWBZP was extracted as the treatment group, so it can be presumed that its therapeutic effect is poor, which, compared with the whole formula of QWBZP, thus led to a continuous decrease in the relative abundance of *Lactobacillus*. *Enterococcus* is one of the common pathogens of infectious diseases and an important pathogen of hospital infections. Compared with other pathogens, *Enterococcus* has unique biological characteristics and is more prone to drug resistance, with serious resistance to the most commonly used clinical antibiotics, so the continued increase in the relative abundance of *Enterococcus* may be related to the resistance shown by *Enterococcus* to antibiotics such as gentamicin. In the LEfSe analysis, there were significant differences in the characteristic bacteria of the mn, mm, and ma groups, further demonstrating the differences in the gut microbial structure of the three groups.

## Conclusion

In summary, antibiotic disrupts the structure of the intestinal mucosal bacteria and reduces their diversity. Our previous study demonstrated the effectiveness of QWBZP in the treatment of AAD. This experiment was conducted by extracting the crude polysaccharide of QWBZP, aiming to investigate the efficacy of QWBZP crude polysaccharide on AAD. The experimental results showed that QWBZP crude polysaccharide helped to restore the diversity, relative abundance, and community structure of intestinal mucosal bacteria to a certain extent. Still, the single crude polysaccharide component was not very effective in treating AAD, so the clinical efficacy of using QWBZP crude polysaccharide in treating AAD needs to be further investigated.

## Data Availability Statement

The datasets presented in this study can be found in online repositories. The names of the repository/repositories and accession number(s) can be found in the article/Supplementary Material.

## Ethics Statement

The animal study was reviewed and approved by Animal Ethics and Welfare Committee of Hunan University of Chinese Medicine.

## Author Contributions

ZT designed the study. CL wrote the manuscript. CL and KZ analyzed the data and performed the experiments. NX, MP, and ZT supervised the work and reviewed the manuscript. The decision to submit the manuscript for publication was made by all the authors. All authors contributed to the article and approved the submitted version.

## Funding

This work was supported by the Natural Science Foundation of Hunan Province (No. 2021JJ40403), the Scientific Research Foundation of Hunan Provincial Education Department (No. 20C1388), the Hunan University of Traditional Chinese Medicine Open Fund for the First Level Discipline of Traditional Chinese Medicine (No. 2020ZYX13), and the Hunan Chinese Medicine First-Class Discipline Project (2018).

## Conflict of Interest

The authors declare that the research was conducted in the absence of any commercial or financial relationships that could be construed as a potential conflict of interest.

## Publisher's Note

All claims expressed in this article are solely those of the authors and do not necessarily represent those of their affiliated organizations, or those of the publisher, the editors and the reviewers. Any product that may be evaluated in this article, or claim that may be made by its manufacturer, is not guaranteed or endorsed by the publisher.
